# A Phase II Trial of Melphalan Based Reduced-Intensity Conditioning for Transplantation of T-Replete HLA-Haploidentical Peripheral Blood Stem Cells with Posttransplant Cyclophosphamide in Patients with Hematologic Malignancies

**DOI:** 10.1155/2021/8868142

**Published:** 2021-03-23

**Authors:** Melhem M. Solh, Gabriel Hinojosa, Justin Laporte, Scott R. Solomon, Lawrence E. Morris, Xu Zhang, H. Kent Holland, Asad Bashey

**Affiliations:** ^1^Blood and Marrow Transplant Program at Northside Hospital, Atlanta, GA, USA; ^2^Center for Clinical and Translational Sciences, University of Texas Health Science Center at Houston, Houston, TX, USA

## Abstract

T-replete haploidentical donor transplants using posttransplant cyclophosphamide (haplo) have greatly expanded donor availability and are increasingly utilized. Haplo were originally performed using truly nonmyeloablative conditioning and a bone marrow graft. We have also developed myeloablative conditioning and peripheral blood stem cell (PBSC) grafts for use with haplo. However, some patients may not tolerate myeloablative conditioning but may still benefit from a more dose-intensified preparative regimen to control malignancy and diminish graft rejection. To this end, we enrolled 25 patients on a prospective phase II trial utilizing a regimen of fludarabine 30 mg/m^2^/day × 5 days and Melphalan 140 mg/m^2^ on day -1 (flu/Mel) followed by infusion of unmanipulated PBSC graft from a haploidentical donor. GVHD prophylaxis included cyclophosphamide 50 mg/kg/day on days 3 and 4, mycophenolate mofetil on day 35, and tacrolimus on day 180. Median age was 57 years (range from 35 to 68). Transplantation diagnosis included AML (*n* = 11), ALL (*n* = 4), MDS/MPD (*n* = 6), NHL/CLL (*n* = 3), and MM (*n* = 1). Using the refined Disease Risk Index (DRI), patients were low (*n* = 1), intermediate (*n* = 13), and high/very high (*n* = 11). 22 out of 25 patients engrafted with a median time to neutrophil and platelet engraftment of 18 days and 36 days, respectively. All engrafting patients achieved full peripheral blood T-lymphocyte and myeloid donor chimerism at day 30. The 180-day cumulative incidence for acute GVHD grades II–IV and III-IV was seen in 20% (95% CI 8%–37%) and 8% (95% CI 2%–22%), respectively. The 2-year cumulative incidence of chronic GVHD was 16% (95% CI 5%–33%) (moderate-severe 12% (95% CI 3%–27%)). After a median follow-up of 28.3 months, the estimated 2-year OS, DFS, NRM, and relapse were 56% (95%CI 33–74%), 44% (95%CI 23%–64%), 20% (95% CI 8%–37%), and 36% (95% CI 17%–55%), respectively. Among patients with high/very high risk DRI, 2-year OS was 53% compared to 69% for low/intermediate DRI. When compared with a contemporaneous cohort of patients at our center receiving haploidentical transplant with nonablative fludarabine, Cytoxan, and total body irradiation flu/Cy/TBI regimen, the outcomes were statistically similar to the 2-year OS at 56% vs. 63% *p*=0.75 and DFS at 44% vs. 46% *p*=0.65.

## 1. Introduction

Allogeneic hematopoietic stem cell transplant (allo-HSCT) is considered a curative modality for many patients with high risk hematologic malignancies. Transplantation using a matched related sibling, if available, is considered the standard choice for patients with high risk malignancies requiring allo-HSCT [1]. Unfortunately, approximately only one-third of candidates for HSCT have HLA-matched siblings. Haploidentical donors are available for most patients as all patients share one haplotype with each of their parents. The use of cyclophosphamide (Cy) postinfusion of stem cells has allowed control of alloreactivity post-haplo without increasing the risk of opportunistic infections as seen using T-cell depletion [2].

The posttransplant cyclophosphamide (PTCy) strategy was originally developed by O'Donnell et al. using a nonmyeloablative conditioning regimen of fludarabine, single fraction 2 Gy TBI, and low-dose pretransplant cyclophosphamide, a non-T-cell-depleted marrow from haploidentical first-degree relatives [2].

The main cause of treatment failure in the nonmyeloablative Hopkins study was relapse, with a cumulative incidence of relapse at 1 and 2 years after transplantation being 50% and 57%, respectively. One explanation for the high rate of relapse, as in other nonmyeloablative HSCT trials, is that the transplantation conditioning used may not be intense enough to achieve sufficient control of malignancy prior to the development of an effective graft-versus-malignancy effect.

In order to reduce the risks of relapse and graft rejection, we have developed approaches to use myeloablative conditioning and PBSC grafts for haplo with PTCY in two consecutive published phase II trials. The first study with busulfan based conditioning established that myeloablative haploidentical transplantation can yield full donor chimerism at day 30, grades III-IV acute GVHD rate of 10%, and a 1-year OS of 69% and DFS of 50% [3]. However, in this study, BK virus induced hemorrhagic cystitis was a significant cause of early posttransplant morbidity. The second myeloablative study with TBI based conditioning showed much lower rates of BK virus hemorrhagic cystitis, full donor chimerism at day 30, a one-year NRM under 10%, and a 2-year DFS of 73% [4]. Despite the success of this approach, a significant limitation of full-intensity myeloablative regimens is that they are poorly tolerated in elderly patients older than 60 years or those with significant comorbidities. There remains a need for a preparative regimen for haplo transplants that is more intense than standard nonablative haplo conditioning (flu/low-dose TBI) but more tolerable than fully myeloablative conditioning regimens as a means of decreasing relapse and preventing graft failure among haplo-HSCT patients.

## 2. Methods

### 2.1. Eligibility and Enrollment

This was a single-center, prospective phase II clinical trial. Written informed consent was obtained for all the patients in accordance with the Declaration of Helsinki. The study was approved by the institutional review board at Northside Hospital. Patients were eligible for inclusion if they were between 18 and 75 years of age, had a hematologic malignancy requiring allogeneic HSCT, were without a readily available matched related or unrelated donor, with an available partially HLA-mismatched haploidentical related donor and adequate organ function as defined by total bilirubin <2 mg/dL, serum creatinine <2 mg/dL, creatinine clearance ≥40 mL/min, left ventricular ejection fraction ≥45%, forced expiratory volume in 1 second, and forced vital capacity ≥50% predicted, Karnofsky performance status ≥70%, and were human immunodeficiency virus negative. Donors were required to be first-degree relatives (parent, child, or sibling) of the recipient and partially HLA matched 3 of 6 to 5 of 6 loci with the recipient. Donors were excluded if they had a positive HLA crossmatch in the host-versus-graft direction or high titer donor-specific antibodies, as determined by the pretransplant panel reactive antibody testing.

### 2.2. Treatment Plan

Transplantation conditioning consisted of fludarabine 30 mg/m^2^/day on days −6 to −2 and melphalan 140 mg/m^2^ on day −1. On day 0, patients received an unmanipulated PBSC allograft with CD34+ dose capped at 5 × 10^6^/kg recipient. On days +3 and +4, patients received cyclophosphamide 50 mg/kg/day plus mesna for bladder protection. On day +5, patients began posttransplantation immunosuppression therapy with tacrolimus 0.03 mg/kg/day (target level 5 to 15 ng/mL) and oral MMF 15 mg/kg three times daily capped at a total daily dose of 3 grams. No systemic immunosuppressive agents were administered until >24 hours after completion of posttransplant cyclophosphamide, including corticosteroids for nausea prevention. MMF and tacrolimus were discontinued without taper at days +35 and +180, respectively. Twice daily ursodiol began 24 hours prior to starting conditioning regimen, day −7, and continued through day +28 for venoocclusive disease (VOD) prophylaxis.

Antimicrobial prophylaxis was administered according to institutional practice guidelines. Standard prophylaxis was started on day 0 including a fluoroquinolone, acyclovir, and an echinocandin. On day +5, antifungal prophylaxis was changed to either oral voriconazole or posaconazole for all patients. Filgrastim 5 *μ*g/kg was given daily starting from day +5 and continued until neutrophil engraftment. All transplants were performed in the outpatient setting with hospital admissions reserved for significant complications.

### 2.3. Study Endpoints

The primary endpoint of this study was to estimate the incidence of graft rejection. Secondary endpoints included overall survival (OS), nonrelapse mortality (NRM), disease-free survival (DFS), as well as the incidence and severity of graft versus host disease (GVHD), and hematologic or nonhematologic toxicities associated with reduced-intensity haploidentical HSCT. Neutrophil engraftment was defined as the first day of three consecutive days after transplantation achieving an absolute neutrophil count >500/*μ*L. Platelet engraftment was defined as the first day of three consecutive days after transplantation with platelet count ≥20,000/*μ*L in the absence of platelet transfusions within the prior 7 days. Nonrelapse mortality was defined as death in the absence of relapse or disease progression.

### 2.4. Chimerism Analysis

PCR-based DNA amplification to detect short tandem repeats was performed to evaluate quantitative chimerism of posttransplant T-lymphocytes and myeloid cells in the peripheral blood. Peripheral blood was analyzed on days +30, +60, +100, and +180, then every six months. Results were reported as the percentage of cells that are of donor origin. Mixed chimerism was defined as the detection of >5% and <95% cells of donor origin, and full donor chimerism was defined as the detection of ≥95% cells of donor origin.

### 2.5. Comparison to Contemporaneous Cohort of Nonmyeloablative Haploidentical Donor Transplantation Patients

A contemporaneous group of haploidentical transplant recipients receiving standard nonmyeloablative conditioning regimen were utilized as a comparator cohort. Sixty-one patients receiving fludarabine, cyclophosphamide, and low-dose TBI (Baltimore regimen) followed by haploidentical transplant were analyzed, and survival outcome results were compared to those of the patients treated on this clinical trial. We also performed a matched-paired comparison analysis for 18 patients in the flu/Mel cohort to 18 matched PBSC graft patients (matched for age range, disease risk, and HCT-CI) in the flu/Cy/TBI contemporaneous cohort.

### 2.6. Statistical Analysis

We conducted Wilcoxon rank sum test to compare median ages of two conditioning regimen groups. Fisher's exact test was used to evaluate associations of categorical patients' characteristics with conditioning regimen. Probabilities of OS and DFS were estimated using the Kaplan–Meier product-limit method. The cumulative incidences of NRM, relapse, acute GVHD, and chronic GVHD were computed to accommodate the presence of competing risks. NRM and relapse were considered as competing risks. Death was considered as competing risk for GVHD endpoints. We computed log-log transformed confidence intervals for survival or cumulative incidence probabilities. Log-rank test was used to compare survival probabilities between two conditioning regimen groups. Gray's test was used to compare cumulative incidences between two conditioning regimen groups. Statistical analysis was performed using the software SAS (version 9.3, the SAS institute).

## 3. Results

### 3.1. Characteristics of the Study Cohort

A total of 25 patients with a median age of 57 years (range from 35 to 68) with high risk hematologic malignancies were enrolled between November of 2015 and December of 2018 (Table 1). Indication for transplantation included acute myelogenous leukemia [5], myelodysplastic syndrome [6], myeloproliferative disorder [3], chronic lymphocytic leukemia [1], acute lymphoblastic leukemia [3], non-Hodgkin lymphoma [6], and multiple myeloma [1]. Using the disease risk index (DRI), patients were classified as low [1], intermediate [7], or high/very high [5]. Twenty patients (80%) had a comorbidity index (HCT-CI) of ≥3. The median inpatient length of stay during the first 100 days was 6 days (range from 1 day to 33 days).

### 3.2. Graft Failure and Engraftment

Three patients (12%) failed to engraft by day 42 with spontaneous count recovery in one of the three patients. Two of the three patients successfully engrafted after their second haplo transplant using different donors. All engrafting patients had sustained complete donor myeloid and T-lymphocyte chimerism by day 30. Median time to neutrophil and platelet engraftment was 18 days and 36 days, respectively.

### 3.3. Acute and  Chronic GVHD

**Figure 1:**
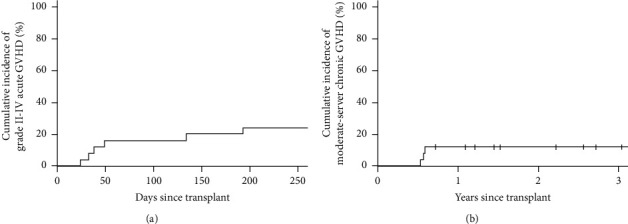
Cumulative incidence of (a) acute GVHD, and (b) chronic GVHD.

The cumulative incidences of grades II to IV and grades III and IV acute GVHD were 20% (95% CI 8%–37%) and 8% (95% CI 2%–22%), respectively (Figure 1(a)). The cumulative incidences of any and moderate-severe chronic GVHD were 16% (95% CI 535%–33%) and 12% (95% CI 3%–27%), respectively (Figure 1(b)).

### 3.4. Adverse Events

Postinfusion fevers as a result of cytokine release syndrome (CRS) from the HLA-mismatched transplant occurred in 23 out of 25 patients (93%). Most CRS cases were of grade 1 (*n* = 21). One patient had a grade 3 CRS, and one patient had grade 2 CRS. In addition to CRS, the most common nonhematologic grades 3/4 adverse events were mucositis (*n* = 9), diarrhea (*n* = 8), and nausea/vomiting (*n* = 2). Severe grade 3 BK cystitis was seen in two patients. CMV reactivation (PCR ≥ 400 IU/ml) was observed in 15 (60%) transplant recipients (83% of CMV-seropositive recipients). No CMV-related disease or mortality was observed. Letermovir was not used for CMV prophylaxis. There were no events of EBV reactivation or EBV associated lymphoproliferative disease.

### 3.5. Nonrelapse Mortality, Relapse, Disease-Free Survival, and Overall Survival

**Figure 2:**
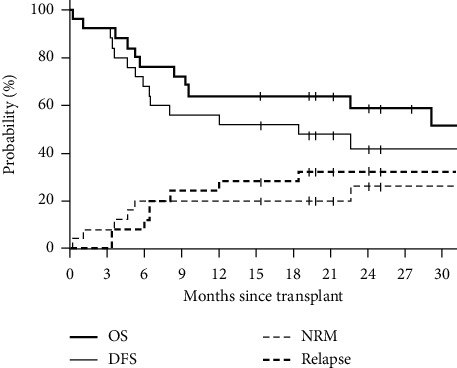
Survival estimates and cumulative incidence of relapse/progression and non-relapse mortality.

After a median follow-up of 28.4 months for surviving patients, the estimated 1- and 2-year overall survival (OS) was 68% (95% CI 46%–82%) and 56% (95% CI 33%–74%), respectively. The corresponding 1- and 2-year DFS was 56% (95% CI 34%–73%) and 44% (95% CI 23%–64%). The cumulative incidence of relapse at 1 year and 2 years was 24% (95% CI 10%–42%) and 36% (95% CI 17%–55%); see Figure 2. The cumulative incidence of nonrelapse mortality was 20% (95% CI 8%–37%) at 2-year posttransplant. GVHD free relapse free survival (GRFS) defined as survival free of grades 3-4 acute GVHD and immunosuppression requiring chronic GVHD was 52% (95% CI 31%–69%) and 41% (95% CI 20%–60%) at 1- and 2-year posttransplant.

For patients with high/very high DRI, the 2-year OS was 53% (31%–69%) compared to 69% (46%–82%) for patients with low/intermediate DRI. For patients with HCT-CI 0–2, the 2-year OS was at 75% (53%–89%) compared to 51% (37%–62%) among patients with HCT-CI ≥3. NRM at 2 years was 20% but was only at 12% for patients age <65 years and 14% for patients with HCT-CI 0–2.

### 3.6. Comparison of Transplantation Outcomes between Nomyeloablative Haplo (flu/Cy/TBI) versus flu/Mel

Outcomes for patients receiving reduced-intensity conditioning regimen (flu/Mel) in this study were compared to a contemporaneous cohort of consecutive patients at our institution receiving nonmyeloablative conditioning (flu/Cy/TBI) (*n* = 61) followed by haploidentical transplant (Table 2). When examining the entire cohort of flu/Mel vs. flu/Cy/TBI, outcomes at 2 years were not significantly different for OS (56% vs. 63%, *p*=0.75), DFS (44% vs. 46%, *p*=0.65), NRM (20% vs. 17%, *p*=0.58), or relapse rate (36% vs. 37%, *p*=0.97). Patients receiving flu/Mel had a lower incidence of acute II-IV GVHD at 100 days (20% vs. 44%, *p*=0.007). Similarly, among 18 matched pairs, there was no difference in graft failure rate (1/18 failed to engraft in each group), overall survival (*p*=0.67), and disease-free survival (*p*=0.65).

## 4. Discussion

Our study reports the outcomes of a conditioning regimen of fludarabine plus melphalan followed by infusion of HLA-mismatched haploidentical peripheral blood stem cells in the setting of posttransplant cyclophosphamide. In this study, we found that flu/Mel based conditioning is feasible, results in sustained engraftment and rapid full donor chimerism, and has low rates of acute and chronic GVHD and similar overall survival and disease-free survival to other reported reduced-intensity and nonmyeloablative regimens [2, 8].

The use of HLA-mismatched haploidentical related donors is now an acceptable standard for patients lacking a suitable donor with outcomes similar to matched related and matched unrelated recipients [9–11]. Most of the HLA-mismatched haploidentical transplants performed in the United States follow the Hopkins regimen with nonmyeloablative flu/Cy/TBI and tend to use marrow as the preferred choice of graft source [5, 12]. The main limitation of nonmyeloablative regimen using marrow grafts remains to be the high rate of relapse especially among high refined disease risk index with a 3-year progression free survival for low, intermediate, and high DRI estimated at 65%, 37%, and 22% [12]. To overcome the high relapse rates, many centers considered peripheral blood stem cells or more intensive conditioning regimens [3, 4]. In a recent analysis from the CIBMTR data base, the use of T-replete HLA-mismatched haploidentical peripheral blood stem cells as graft source was found to result in similar engraftment (93% vs. 88%, *p*=0.07), higher risk of GVHD, and lower risk of relapse compared to bone marrow grafts [13]. Higher risk of relapse after BM was limited to patients with leukemia (HR 1.49, *p*=0.009). Another analysis from the CIBMTR compared outcomes of HLA-mismatched haploidentical transplant recipients after a myeloablative conditioning to reduced-intensity conditioning [7]. In this analysis, for young patients (18–54 years), disease-free survival was lower (42% vs. 51%; *p*=0.007), and relapse was higher (44% vs. 33%, *p*=0.001) with a reduced intensity regimen compared with a myeloablative regimen. Although there was a lower relapse beyond 2 years after transplant, no difference in PFS was seen among older patients based on conditioning intensity, and this was hypothesized to be related to the high early NRM with myeloablative regimens for this patient population.

The question of conditioning intensity and outcomes after allogeneic HCT has been addressed in multiple single-center analysis [14] and randomized clinical trials [15, 16]. In most of these studies, myeloablative conditioning resulted in better disease control compared to reduced intensity especially for acute myeloid leukemia and MDS.

Our study used fludarabine/melphalan followed by HLA-mismatched haploidentical peripheral blood stem cells with posttransplant cyclophosphamide in an attempt to establish a regimen that has more intensity than commonly used nonmyeloablative flu/Cy/low-dose TBI and can be well tolerated in older patient population and/or those younger patients who cannot tolerate a full-intensity myeloablative ablative regimen. This regimen was well tolerated, had sustained engraftment with full chimerism, and could be safely administered in patients up to age 68 years and with significant comorbidities. There were four patients with myeloproliferative disease in this study, and engraftment was achieved in 3 out of three patients with myelofibrosis. The acute and chronic GVHD rates seen with this regimen are in line with reported data from other haploidentical results using posttransplant cyclophosphamide. A comparison to the nonmyeloablative regimen in our center showed similar results; however, this was not a matched control comparison, and there were multiple differences in the two cohorts that can affect the survival and disease-free survival endpoints such as high risk disease distribution and worse HCT-CI in the flu/Mel population. In a matched controlled comparison, the outcomes were also similar, though the numbers were not large enough to identify any significant differences.

The toxicity profile of flu/Mel with T-replete haploidentical peripheral blood stem cells showed a very high rate of cytokine release syndrome; however, most of these cases (91%) were of grade I and did not require additional interventions besides our routine supportive care. Graft failure with this regimen is more of a concern than graft failure after nonablative regimen since autologous recovery is unlikely to occur after a more intensive regimen such as flu/Mel. The other common toxicities of grade 3 mucositis and gastrointestinal adverse events make flu/Mel with posttransplant cyclophosphamide a challenging regimen to give for older patients, and we recommend limiting the use of such regimen to patients younger than 70 years old. The high mucositis rate is related to the use of high dose melphalan with the combination of high dose cyclophosphamide within 5 days. A further study with reduction in Melphalan dose to 100 mg/m^2^ is being planned.

## Figures and Tables

**Table 1:** Patients' characteristics (*N* = 25).

	*N* = 25
Age, median (range)	57 (35, 68)
Male sex	11 (44%)
Diagnosis	
AML	11 (44%)
ALL	4 (16%)
MDS/MPS	6 (24%)
NHL/CLL	3 (12%)
PCD	1 (4%)
Patient CMV	
Positive	18 (72%)
Negative	7 (28%)
ABO compatibility	
Compatible	16 (64%)
Incompatible minor	2 (8%)
Incompatible minor and major	1 (4%)
Incompatible major	6 (24%)
DRI	
Low	1 (4%)
Intermediate	13 (76%)
High/very high	11 (16%)
CMl	
1–3	11 (44%)
4–8	14 (56%)
Female donor male recipient	4 (16%)
Year of transplantation	
2015	3 (12%)
2016	8 (32%)
2017	6 (24%)
2018	8 (32%)
Number of survivors	14
Survivor follow-up (month), median (range)	28.3 (8.7, 43.9)

**Table 2:** Patients' characteristics in two groups.

*p* value	flu/Mel	flu/Cy/TBI	
*N*	25	61	
Age, median (range)	57 (35, 68)	61 (22, 75)	0.10
Male sex	11 (44%)	32 (52%)	0.64
Diagnosis			0.08
AML	11 (44%)	13 (21%)	
ALL	4 (16%)	4 (7%)	
MDS/MPS/CML	6 (24%)	14 (23%)	
NHL/CLL/HD	3 (12%)	24 (39%)	
PCD	1 (4%)	2 (3%)	
Other	0 (0%)	4 (8%)	
Cell source			<0.001
PBSC	25 (100%)	37 (61%)	
BM	0 (0%)	24 (39%)	
Patient CMV			0.38
Positive	18 (72%)	50 (82%)	
Negative	7 (28%)	11 (18%)	
ABO compatibility			0.32
Compatible	16 (64%)	33 (54%)	
Incompatible minor	2 (8%)	15 (25%)	
Incompatible minor and major	1 (4%)	2 (3%)	
Incompatible major	6 (24%)	11 (18%)	
DRI			0.06
Low	1 (4%)	7 (12%)	
Intermediate	13 (52%)	27 (45%)	
High/very high	11 (44%)	23 (38%)	
CMl			0.09
1–3	11 (44%)	39 (64%)	
4–8	14 (56%)	22 (36%)	
Female donor male recipient	4 (16%)	8 (13%)	0.74

## Data Availability

The data set used in the study is available in our institutionalized database.
